# A reply to analysis of static parameters in retrospective studies: limitations and interpretation

**DOI:** 10.1186/s13054-023-04709-x

**Published:** 2023-11-02

**Authors:** Peter J. McGuigan, Elisa Giallongo, Alistair D. Nichol, Markus B. Skrifvars, Daniel F. McAuley

**Affiliations:** 1 Regional Intensive Care Unit, Royal Victoria Hospital, Belfast, UK; 2 Wellcome-Wolfson Institute for Experimental Medicine, Queen’s University Belfast, Belfast, UK; 3 Intensive Care National Audit & Research Centre, Napier House, 24 High Holborn, London, UK; 4 University College Dublin Clinical Research Centre, St Vincent’s University Hospital, Dublin, Ireland; 5 The Australian and New Zealand Intensive Care Research Centre, Monash University, Melbourne, Australia; 6 The Alfred Hospital, Melbourne, Australia; 7 Department of Emergency Care and Services, University of Helsinki, Helsinki University Hospital, Helsinki, Finland

We thank Dr. Shen for their interest in our study [1, 2]. Dr. Shen raises important points on the effect of critical care interventions on blood pressure, the impact of blood pressure exposure over time on outcomes, and the clinical implications of our study [2].

The physiological data contained within the Case Mix Programme in our study are collected by trained data collectors according to strict rules set out in a Data Collection Manual [1]. These rules state blood pressure values “… *should not be recorded for any admission during periods of iatrogenic disturbance, e.g. physiotherapy, turning, periods of crying, *etc*.”* Therefore, the critical care interventions described by Dr. Shen will not have contributed to highest and lowest blood pressure recordings in our study [1, 2].

Dr. Shen argues that recording extremes of blood pressure may not accurately reflect blood pressure exposure over time. Whilst we agree, how blood pressure exposure is modelled in observational studies should be considered [3]. Importantly, there appears to be no association between the arithmetic mean of blood pressure over time and outcomes following cardiac arrest [4]. This may occur as periods of hypertension numerically average out periods of hypotension. Laurikkala and colleagues found an association between lowest recorded mean arterial pressure (MAP) in the first 6 h and mortality but no association between time-weighted average MAP (TWA-MAP) and mortality [5]. Kilgannon and colleagues found no association between TWA-MAP and neurological outcome [4]. Chui and colleagues used an integral of blood pressure and time below a MAP threshold of 65 mmHg to measure exposure to hypotension. They found that this area below a threshold was associated with mortality, whereas mean MAP was not [6]. Whilst we acknowledge that the inability to present time-weighted average blood pressure is a weakness of our study, exposure to extremes of blood pressure is consistently associated with harm.

Finally, Dr. Shen poses the question of how to manage a patient with a MAP ranging from 75 to 80 mmHg considering our findings [1]. Skrifvars and colleagues conducted a Beyesian model meta-analysis of randomized controlled trials to predict the likelihood of future trials detecting a benefit with a higher MAP target following cardiac arrest. The posterior probability of achieving at least a 5% reduction in mortality or poor neurological outcome was < 50% using a non-informative prior. Using an informative pessimistic prior, the posterior probability fell to < 0.5%. Thus, the authors could not exclude a risk of harm, leading them to conclude that “…*caution must be exercised before deviating from the current guideline recommendation of MAP* > *65 mmHg*…” [7]. In our study, crossing a lower threshold of < 60 mmHg or an upper threshold > 104 mmHg was associated with increased mortality [2, 8]. These findings are largely consistent with recommendations to avoid MAP < 65 mmHg and MAP > 100 mmHg made by the European Resuscitation Council and International Liaison Committee on Resuscitation [9, 10]. We agree with Dr. Shen that more randomized controlled trial evidence is required before deviating from current guidelines. The results of the Sedation, Temperature, and Pressure After Cardiac Arrest and Resuscitation (STEPCARE) trial (NCT05564754) will help inform these outstanding questions.

## Data Availability

Not applicable.

## Abbreviations

MAP: Mean arterial pressure
TWA-MAP: Time-weighted average mean arterial pressure
